# Study on the relationship between scoliosis and vision problems: A narrative review

**DOI:** 10.1097/MD.0000000000035178

**Published:** 2023-10-20

**Authors:** Yingsen Pan, Haoyang Zhang, Xin Ye, Shuailin Li, Xiaoming Li, Zengtu Li, Xiaoming Ying

**Affiliations:** a The 3rd Clinical Medical College of Zhejiang Chinese Medical University, Hangzhou, China; b Tuina Department, The 3rd Affiliated Hospital of Zhejiang Chinese Medical University, Hangzhou, China.

**Keywords:** horizontal gaze, myopia, paralysis, scoliosis, vision loss

## Abstract

Adolescent scoliosis is one of the most common surgical disorders of the pediatric spine. With timely detection and early treatment, most scoliotic children can avoid major and expensive surgery. Vision problems are also frequently found at an early age and can take a toll on individuals quality of life. However, scoliosis, a severe health hazard to adolescents, is often accompanied by vision problems clinically, including myopia, astigmatism, strabismus, amblyopia, horizontal paralysis, and blindness. And people with genetic defects have a higher probability of suffering both spinal problems and vision problems than those with nongenetic defects. However, many individuals viewed scoliosis and vision problems as 2 irrelevant diseases. This review searched PubMed, China National Knowledge Infrastructure, and Web of Science for studies on adolescent, scoliosis, eye diseases, myopia, strabismus, spinal disorders, and vision problems for almost 3 decades, and thus confirmed the potential relationship between adolescent scoliosis and vision problems.

## 1. Introduction

Scoliosis refers to a 3-dimensional deformity characterized by lateral curvature of the spine in the coronal plane, deviation in the sagittal plane, and rotation in the axial plane.^[1]^ Idiopathic scoliosis (IS), the most common type of scoliosis, can be divided into infantile type, juvenile type, adolescent type, and adult type chronologically, among which, adolescent idiopathic scoliosis (AIS) is the most common spinal deformity, affecting 1% to 4% of adolescents, with the fastest progression during puberty.^[2]^ The survey showed that the number of students with scoliosis at primary and secondary schools exceeded 5 million and increased to approximately 300,000 per year in China.^[3]^ Untreated scoliosis can lead to extremely severe cardiopulmonary diseases and even death.^[4]^ Myopia, as the leading cause of visual impairment worldwide,^[5]^ bothered more than half of Chinese teenagers according to the research.^[6]^ And along with scoliosis, it has also been listed as one of the top 3 diseases endangering the health of Chinese adolescents.^[7]^ Individuals often show abnormal posture during vision screening.^[8]^ In addition, some researchers have found that patients with scoliosis also have myopia.^[9]^ This phenomenon may indicate a potential link between scoliosis and vision problems. But many people tend to view scoliosis and vision problems as 2 irrelevant diseases as the former is an orthopedic disease, and the latter, such as myopia and horizontal gaze paralysis, are ophthalmic diseases.^[10,11]^ However, in recent years, researchers found that scoliosis might be related to ophthalmic torticollis, horizontal gaze palsy, Goldenhar syndrome, and so on.^[12]^ Accordingly in the study, we aimed to verify the correlation between scoliosis and various vision problems.

## 2. Methods

### 2.1. Search strategy

A literature search was conducted in PubMed, China National Knowledge Infrastructure, and Web of Science. These databases are indexed according to strict selection criteria, and the articles are high quality with reliable data sources. The search items are as follows: scoliosis odds ratio (OR) spinal disorders AND teenagers OR adolescent AND vision disorders OR myopia OR vision problems OR eye diseases OR strabismus.

### 2.2. Selection criteria

We included articles; Related to adolescent scoliosis and vision problems; Available as published full text, and; With specific language (abstract in Chinese or English) and time frame. We excluded studies; Lack of key information and; With duplicate publications or dates.

### 2.3. Data collection and analysis

**Figure 1.**
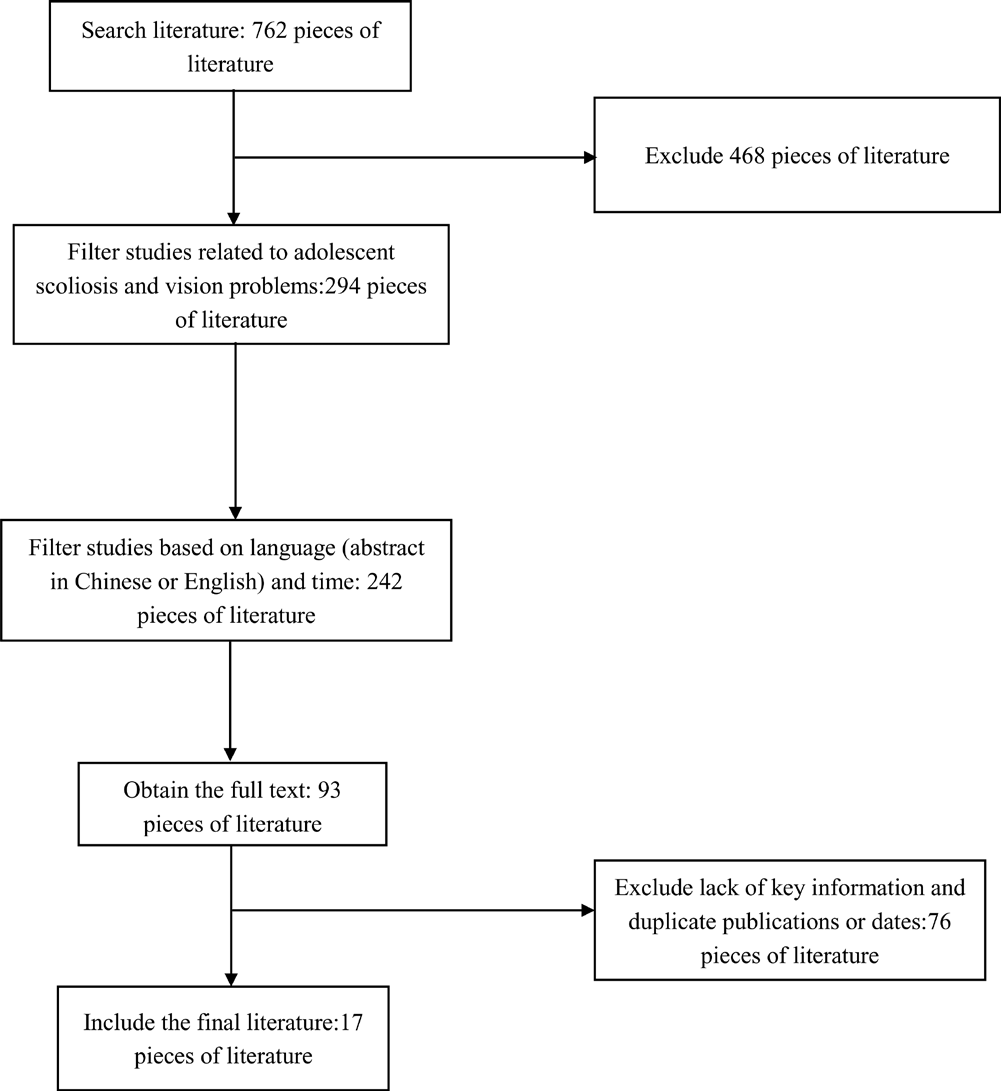
Flowchart of study selection.

Two review authors independently screened all data identified in the searches. The literature on the correlation between adolescent scoliosis and various vision problems is presented in Table 1. The flowchart of study selection is shown in Figure 1.

**Table 1:** The literature about the correlation between scoliosis and various vision problems.

No.	Author(s)	Publication year	Main content	Vision disease	Conclusion
1	Catanzariti, Jean F. Salomez, Elisabeth Bruandet, Jean M.	2001	Studied the relationship between visual defects and scoliosis	Visual impairment	The prevalence of scoliosis is higher in children with visual impairment.
2	Cai Z, Wu R, Zheng S, Qiu Z, Wu K.	2021	A morphological and epidemiological study of idiopathic scoliosis in primary school students in Chaozhou city.	Myopia	The occurrence of myopia and scoliosis is correlated.
3	Zhang Lifang, Deng Shuzhen, Liu Chunyan, et al	2021	The occurrence of suspected symptoms and related influencing factors in idiopathic scoliosis (AIS).	Myopia	The uncorrected visual acuity were factors in the occurrence of.
4	Egorova TS, Smirnova TS, Romashin OV, Egorova IV.	2016	The possibility of vertebral deformity and its correction in children with a high-degree of complex myopia.	High-degree complex myopia	The spinal deformities are more pronounced in visually impaired students with high myopia.
5	Ulusoy DM, Akkaya S, Batin S.	2020	The application of enhanced depth imaging optical coherence tomography in adolescent idiopathic scoliosis.	Choroidal thickness	Choroidal thickness is lower with adolescent idiopathic scoliosis.
6	Kim JH, Yum TH, Shim JS.	2019	Effective treatment for secondary cervicothoracic scoliosis in patients with torticollis	Ocular torticollis	Scoliosis frequently occurs in patients with torticollis, but the relationship between the two conditions requires further study.
7	Xiu Y, Lv Z, Wang D, Chen X, Huang S, Pan M.	2021	Study on a novel ROBO3 mutation in patients with horizontal gaze palsy with progressive scoliosis in Chinese families.	Horizontal gaze palsy	The ROBO3 gene causes the simultaneous occurrence of scoliosis and visual disorders.
8	Shalini P, Shah VM.	2017	A case report about Horizontal gaze palsy with progressive scoliosis.	Horizontal gaze palsy	HGPPS is characterized by defective vision and progressive scoliosis.
9	Dolar Bilge A.	2019	A 12-year-old boy, who was admitted for scoliosis surgery, has also suffered from horizontal gaze palsy since birth.	Horizontal gaze palsy	Horizontal gaze palsy and progressive scoliosis are the main findings of the HGPPS.
10	Ucan B, Kaynak Sahap S, Bako D, Tiras ST, Ceylaner S, Fitöz S.	2022	The MRI and DTI findings of an infant with ocular findings but no scoliosis.	Horizontal gaze palsy	Patients with horizontal gaze palsy with progressive scoliosis may only have ocular disease but not have scoliosis.
11	Khan AO, Abu-Amero K.	2014	Eye-related disorders in patients with horizontal gaze palsy accompanied by progressive scoliosis.	Strabismus	Horizontal gaze palsy with progressive scoliosis can lead to the strabismus.
12	Pan XX, Huang CA, Lin JL, et al	2020	The epidemiology of thoracic scoliosis in children and adolescents with strabismus the association of two diseases.	Strabismus	The type of strabismus and degree of strabismus showed a significant relationship with the prevalence of thoracic scoliosis.
13	Sharma N, Passi S.	2013	Discussed Goldenhar syndrome.	Ocular developmental abnormalities	Scoliosis and ocular diseases have a reciprocal influence on each other.
14	Grossman W, Ward WT.	1993	Central retinal artery occlusion after scoliosis surgery with a horseshoe headrest. Case report and literature review.	Visual loss	Vision loss due to scoliosis surgery.
15	Shillingford JN, Laratta JL, Sarpong NO, et al	2018	To investigate visual-related complications in spinal deformity patients undergoing spine surgery.	Visual loss	Scoliosis surgery carries a risk of vision loss.
16	De la Garza-Ramos R, Samdani AF, Sponseller PD, et al	2016	To investigate nationwide estimates of POVL after corrective surgery for pediatric scoliosis.	Visual loss	The incidence of Visual loss was estimated at 0.16%.
17	Yin XJ, Xie WN, Si JY.	2005	A patient with scoliosis was blinded after spinal surgery	Visual loss	Surgery in the prone position is a risk factor for visual loss.

AIS = adolescent idiopathic scoliosis, HGPPS = horizontal gaze palsy with progressive scoliosis.

## 3. Results

### 3.1. Scoliosis and myopia

Scoliosis is a 3-dimensional deformity with disordered sequences in the coronal, sagittal, and transverse planes of the spine.^[13]^ In general, the diagnostic standard is a Cobb angle > 10° in the coronal plane.^[14]^ IS is the most common type of scoliosis. Myopia refers to blurred vision when the light entering the eyeball is focused in front of the retina.^[15]^ According to 2018 data, the myopia rate among Chinese adolescents was 53.6%, with middle and high school students accounting for the highest proportion of myopia and females outnumbering males.^[16]^ Women were also more likely to have scoliosis than men.^[17]^ Zeming Cai et al^[18]^ conducted a study on the incidence of scoliosis among more than 5000 students in Chaozhou City, China, and the multiple logistic regression analyses showed that the female sex (OR = 2.45) and myopia (OR = 1.49) were significantly associated with scoliosis. Furthermore, nearsighted students were 1.49 times more likely to develop scoliosis than those without myopia. A study on the influencing factors of IS in high school in Yunnan Province, China, found that gender, age, and uncorrected visual acuity were important factors in the occurrence of AIS; the study also pointed out that relatively poor vision may lead to a decrease in outdoor activities and an increase in sedentary time, which could also be one of the reasons for the suspected symptoms of AIS.^[19]^ That was to say, some poor habits, such as staring at screens for long periods and holding books too close in daily life, may aggravate scoliosis. Egorova et al^[20]^ after checking pediatric patients with high myopia complications and assessing their spinal morphology, revealed that various deformations of the musculoskeletal system, including scoliosis, were particularly evident in patients with high myopia compared to controls.

Myopia is an irreversible eye disease caused by the continuous stretching and degeneration of the choroid, sclera, and retina.^[21]^ Stephen et al^[22]^ reported that the subfoveal choroid was significantly thinner in myopic patients concerning the choroid and retinal thickness. This study could not determine whether choroidal thinning in myopic patients was caused by passive thinning of the vitreous cavity during myopia development or by the asymmetry of different light signals between the eyes. Recently, Ulusoy et al^[23]^ assessed choroidal thickness in AIS with enhanced depth imaging optical coherence tomography and noticed that the mean subfoveal choroidal thickness in patients with scoliosis was lower than that in a control group. The difference was also significant at all survey points outside the central concave area (*P* < .001). This study provides a preliminary conclusion that the choroidal thickness in patients with scoliosis is lower than that in healthy individuals. Accordingly, through the change in choroidal thickness, we could see that there was a certain correlation between scoliosis and myopia, but these data were not universal. Further observations and studies are required.

### 3.2. Scoliosis and ocular torticollis

Torticollis is a common disease in children with increased morbidity.^[24]^ It results from sternocleidomastoid muscle spasms characterized by involuntary head tilting. Torticollis is divided into true and false torticollis.^[25,26]^ False torticollis is unrelated to the sternocleidomastoid muscle; it is caused by extraocular muscle problems with diplopia, and the abnormal head position is to maintain binocularity. Therefore, it is also known as ocular torticollis.^[27]^ Although secondary scoliosis often occurs in patients with torticollis, research on the relationship between scoliosis and torticollis is rare.^[28]^ As a result, ocular torticollis is often overlooked in advanced stages and misdiagnosed as scoliosis only.

Most previous scoliosis studies have focused on the thoracic and lumbar spinal segments,^[29]^ meanwhile, indicating inadequate assessment of the segmental effects of scoliosis on the cervical and caudal vertebrae. If the cervical vertebrae, which are located at the top of the spine, are in an abnormal position, then the sequence of the entire spine is disordered.^[30]^ Existing studies have found that cervical curvature changes can lead to thoracic spinal compensation and further result in lateral curvature.^[31]^ The original symptoms caused by eye abnormalities tend to weaken over time because of compensatory body posture, leaving only scoliosis, headaches, and back pain.^[32]^ As a result, many doctors often treat in the wrong site while ignoring problems with ocular muscles, causing unnecessary pain and suffering to patients.

### 3.3. Scoliosis and horizontal gaze palsy

Horizontal gaze palsy with progressive scoliosis (HGPPS) is an autosomal recessive genetic disease caused by mutations ROBO3.^[33]^ It was first reported by Sharpe et al^[34]^ in 1975 and was found to be caused by a mutation in ROBO3 more than 30 years later. The main clinical features of HGPPS include congenital horizontal gaze palsy (absence of horizontal eye movement), progressive scoliosis, and ametropia. Imaging showed incomplete brain bridge development, no facial depression, a medullary pterygoid, and a deep cleft in the midline of the cerebral bridge.^[35]^ It occurs primarily during childhood and adolescence.^[36]^ ROBO3 regulates the midline crossing of the posterior axons of the brain. Therefore, Dolar Blige^[37]^ speculated that horizontal gaze palsy might be related to abnormal inputs in the outer abducent nucleus of the pontine reticular structure and the inability of axons to develop in the medial longitudinal bundle to cross the midline. Moreover, ROBO3 mutations are also thought to cause musculoskeletal changes during the development of scoliosis.

One study reported that a Serbian child had all clinical and neuroimaging characteristics of HGPPS; however, no ROBO3 mutation was found.^[38]^ After DNA extraction and gene sequencing, Sami et al^[39]^ discovered the effects of cytoplasmic domains C2 and C3 on the pathological appearance of HGPPS and emphasized that inbreeding increased the risk of disease. Other scholars have also proposed similar hypotheses. Scoliosis is a more pronounced external manifestation of the disease than ocular symptoms.^[40]^ However, in young patients, ocular symptoms prevail while scoliosis can be difficult to detect, resulting in delayed diagnosis and treatment. Pediatric strabismus is a common ophthalmic condition derived from the dysfunction of ocular muscle coordination and is manifested as the inability of both eyes to focus simultaneously on the same object.^[41]^ According to the presence or absence of eye movement disorders, strabismus can be divided into commonality and non-commonality; HGPPS is part of the differential diagnosis of the latter.^[42]^ Pan XX and colleagues,^[43]^ in a study of 1024 male and 911 female patients with an average age of 8.55 ± 4.31 years undergoing strabismus surgery, found that the incidence of thoracic scoliosis was more common in patients aged 7 to 9 years old and those with exotropia. However, the prevalence of thoracic scoliosis should also consider gender and type of strabismus. The study is limited by the inclusion of only patients undergoing strabismus surgery without further subdivision, which introduces bias and confounding factors. The key to treatment is to understand the pathogenesis and clinical manifestations of the disease and identify the truth regarding the disease.

### 3.4. Goldenhar syndrome

Goldenhar syndrome gets its name from Dr Maurice Goldenhar, a French researcher who revealed this eye-, ear-, and face-related disorder in 1952.^[44]^ In 1963, Gorlin et al^[45]^ discovered a spinal abnormality related to the disease, and in 1989, Cohen changed the name to “eye-ear-spine syndrome”. Typical symptoms of the disease include ocular dermoid cysts, strabismus, epicanthus, small or accessory ears, and scoliosis. The pathogenesis of Goldenhar syndrome is not clear; however, the eye, ear, and spinal defects caused by Goldenhar syndrome are mostly due to the abnormal development of the first and second branchial arches, the first branchial fissure, and the temporal bone.^[46]^ In addition, some researchers believe that there is no separation between the ectoderm and the subsequent mesoderm in the early stages of embryonic development in patients with Goldenhar syndrome, which leads to the occurrence of spinal symptoms.^[47]^ Goldenhar syndrome is rare in clinics and has been rarely reported in the Chinese literature. It is important to note that not all patients with Goldenhar syndrome will have concurrent spinal scoliosis and visual impairments.^[48]^ Many people visit doctors only for eye problems, however, these associated orthopedic abnormalities, including scoliosis, horseshoe varus, and congenital hip dislocation, are also present but less noticed. The discovery of Goldenhar syndrome reveals the correlation between scoliosis and ocular diseases, but the potential causes of its occurrence and development require further investigation.

### 3.5. Spinal surgery and vision loss

Eye injuries and vision loss are extremely rare complications of spinal surgery.^[49]^ The incidence of visual loss after spinal surgery ranges from 0.028% to 0.2%, with scoliosis correction as the primary surgery.^[50]^ At the end of the last century, Qiuxu Wang et al^[51]^ reviewed more than 30 spinal surgery cases complicated by visual impairment and found that vision loss after spinal surgery was rare for complex reasons. However, as the number of spinal surgeries and incidence of postoperative blindness has increased, spinal surgeries have surpassed cardiac surgery as the leading cause of postoperative blindness.^[52]^ Data compiled by the scoliosis research society on surgeon-reported postoperative complications showed that from 2009 to 2012, the incidence of visual complications was approximately 12.5 per 100,000 patients with spinal deformities. And the incidence of kyphosis surgery was much higher than that of scoliosis and spondylolisthesis surgeries.^[53]^ Young and male patients were more likely to experience vision loss after surgery than other age groups and female patients.^[54]^ The types of postoperative vision diseases included external eye injury, cortical blindness, retinal ischemia, ischemic optic neuropathy, and acute glaucoma after the comprehensive study of all articles related to vision loss after spinal surgery.^[55]^ Among them, ischemic optic neuropathy occurred most frequently.^[56]^ Especially during lumbar spine surgery.^[57]^

The underlying mechanism of vision loss after spinal surgery remains to be elucidated in detail. It has been found that in some cases, excessive fluid replacement may lead to elevated intraocular pressure, optic nerve effusion, or both in patients undergoing spinal surgery in the prone position.^[58]^ Although there is no direct evidence that fluid replacement leads to postoperative vision loss, there is no doubt that it can dilute the blood, causing intraoperative anemia and hypoxic damage to the optic nerve. Moreover, it has also been reported that the use of vasopressors, such as deoxy adrenaline and epinephrine during spinal surgery increased the likelihood of vision loss.^[59]^ However, since the mechanism of action of vasopressors in this disease is not clear, no definite inference can be drawn at present. Another research conducted by Yanhui Cui et al^[60]^ discovered that the patient’s eyeball was prone to compression during spinal surgery, resulting in visual impairment. However, this study did not rule out the possibility of vision loss when the eyeball was not compressed. Yin et al^[61]^ suggested that prolonged prone use of a horseshoe headrest during spinal surgery was responsible for blindness and that increased intraocular pressure became the risk of postoperative complications. Another survey summarized that fluid replacement, vasoconstrictor use, elevated venous pressure, hypotension, long-term prone position, head position, blood loss, anemia, and patient-specific vascular susceptibility were risk factors for postoperative blindness.^[62]^ And in spinal surgery, these risk factors may not exist alone, which undoubtedly increases their risk of occurrence.^[63]^ Despite medical advances, the risk factors associated with serious complications, such as spinal surgery blindness, require comprehensive and detailed discussion. However, the causes of vision loss are complex and effective treatments are limited. The best way to avoid vision loss after spinal surgery is to ascertain the etiology and reduce these various risk factors.

## 4. Conclusion and prospect

The correlation between scoliosis and vision problems has been demonstrated according to previous studies, but the specific mechanism still needs further exploration. The case-control study, genetic study, and long-term follow-up should be employed in future studies so that the underlying mechanism and relationship between scoliosis and vision problems can be elucidated clearly.

## 5. Limitations

There are some limitations of our study, including the following points. First, this study did not include all the relevant literature. Second, there are some differences between the results of bibliometric analysis and actual research because of the low number of citations in published research.

## Acknowledgments

This work was supported by the Traditional Chinese Medicine Science and Technology Project of Zhejiang Province, China (Grant No. 2022ZA088) and Key Discipline Project of High-level TCM of National Administration of Traditional Chinese Medicine (GJXK2023-85).

## Author contributions

**Conceptualization:** Xiaoming Ying.

**Funding acquisition:** Xiaoming Ying.

**Supervision:** Xiaoming Ying.

**Writing – original draft:** Yingsen Pan.

**Writing – review & editing:** Haoyang Zhang, Xin Ye, Shuailin Li, Xiaoming Li, Zengtu Li.
